# Supplementary material for: Microbial Virulence Factors, Antimicrobial Resistance Genes, Metabolites, and Synthetic Chemicals in Cabins of Commercial Aircraft
Source: Metabolites. 2023 Feb 24;13(3):343. doi: 10.3390/metabo13030343 (PMC10058785; doi:10.3390/metabo13030343)
Supplement: Supplementary file 1 [file metabolites-13-00343-s001.zip › metabolites-2238067-supplementary.pdf]

Table S1. Enriched Virulent Factors in TSC and LSC. The table displays virulent factors that are significantly enriched (LDA > 2, p-value < 0.01 from LEfSe analysis and fold changes > 1.5 or < 0.67) in TSC and LSC. The average reads coverage was calculated as the abundance of virulent factors. To determine the potential microbial sources, all sequences were searched against the NCBI-NT databases. Three microbial species with the highest abundance were identified as the main sources of the virulence factors in the aircraft.

Aircraft	VF category	Subcategory or features	VF name	Abundance in TSC	Abundance in LSC	TSC/LSC fold changes	p-value	Mainly derived microbial species
TSC	Adherence	Non-fimbrial adherence	CBPs	2.9829	1.3428	2.22	0.0005	*Streptococcus mitis*, *Streptococcus pneumoniae*, *Streptococcus pseudopneumoniae*
		Autotransporter adhesin	Hia/Hsf	2.3602	0.6836	3.45	0.0003	*Haemophilus parainfluenzae*, *Achromobacter xylosoxidans*, *Neisseria elongata*
			BadA/Vomp	1.9006	0.6462	2.94	0.0003	*Veillonella parvula*, *Stenotrophomonas maltophilia*, *Agrobacterium tumefaciens*
			BoaB	1.9263	0.7361	2.62	0.0003	*Veillonella parvula*, *Haemophilus parainfluenzae*, *Xanthomonas campestris*
			BoaA	0.6933	0.2776	2.5	0.0005	*Veillonella parvula*, *Haemophilus parainfluenzae*, *Xanthomonas campestris*
		Surface-associated adhesin	C5a peptidase (*Streptococcus pyogenes*)	0.9895	0.5207	1.9	0.0003	*Gardnerella vaginalis*, *Finegoldia magna*, *Rothia mucilaginosa*
			C5a peptidase (*Streptococcus agalactiae*)	1.0756	0.6258	1.72	0.0031	*Finegoldia magna*, unidentified *Hymenobacter*, *Streptococcus sanguinis*
		Cell wall anchored protein	AS	0.504	0.1633	3.09	0.0003	*Streptococcus salivarius*, *Streptococcus sanguinis*, *Azospirillum brasilense*
			Lmb	0.8011	0.4536	1.77	0.0071	*Streptococcus sanguinis*, *Lactococcus lactis*, *Finegoldia magna*
		Type IV pili	Type IV pili	1.521	0.9975	1.52	0.0023	unidentified *Massilia*, *Variovorax paradoxus*, *Stenotrophomonas maltophilia*
	Biofilm	Quorum sensing	Fsr	0.643	0.2851	2.26	0.0009	*Streptococcus parasanguinis*, *Streptococcus mitis*, *Staphylococcus epidermidis*
			AI-2	0.6907	0.403	1.71	0.0017	*Corynebacterium aurimucosum*, *Brachybacterium faecium*, *Propionibacterium acnes*

	Exoenzyme	Protease	Neuraminidase	1.5796	0.5049	3.13	0.0005	*Streptococcus mitis*, *Streptococcus pneumoniae*, *Streptococcus oralis*
	Immune modulation	Lipooligosaccharide	LOS (*Haemophilus influenzae*)	3.4053	2.2482	1.51	0.0054	unidentified *Hymenobacter*, *Veillonella parvula*, unidentified *Massilia*
		Immunoglobulin protease	IgA1 protease	3.2216	0.8907	3.62	0.0003	*Streptococcus pneumoniae*, *Streptococcus mitis*, *Streptococcus gordonii*
	Nutritional/Metabolic factor	Iron uptake	Isd	1.0616	0.6614	1.61	0.0091	unidentified *Eubacterium*, *Coprococcus catus*, *Staphylococcus aureus*
		Carbohydrate uptake	Hpt	0.9551	0.5365	1.78	0.0041	*Klebsiella pneumoniae*, *Rothia mucilaginosa*, *Veillonella parvula*
LSC	Adherence	Attachment organelle	Cytadherence organelle	0.9559	1.5034	0.64	0.0092	*Corynebacterium aurimucosum*, *Corynebacterium testudinoris*, *Brevibacterium linens*

Table S2. Enriched antibiotic resistance genes in TSC and LSC. Only significantly enriched ARGs with LDA > 2, p-value < 0.01 (as determined by LEfSe analysis) and abundance fold changes > 1.5 or < 0.67 are displayed. The abundance of ARGs was calculated as the average reads coverage. The potential microbial sources for each ARG were identified by searching its sequence against the NCBI-NT databases. The two microbial species with the highest abundance were considered as the main source of the ARG in the aircraft and are listed in the table.

Group	ARG category	ARG Name	AMR Gene Family	abundance TSC	abundance LSC	TSC/LSC fold changes	p-value	Mainly derived microbial species
LSC	antibiotic target alteration	vanSF	glycopeptide resistance gene cluster;vanS	0.7263	1.2125	0.6	0.0031	*Variovorax paradoxus*, unidentified *Massilia*, unidentified *Sphingomonas*
		vanRA	glycopeptide resistance gene cluster;vanR	0.3509	0.5801	0.6	0.0092	unidentified *Massilia*, *Paracoccus denitrificans*, *Variovorax paradoxus*

antibiotic efflux	QepA1	major facilitator superfamily (MFS)	0.3906	0.6529	0.6	0.0023	*Nocardioides sp. JS614*, *Methylobacterium radiotolerans*, unidentified *Curtobacterium*
	mtrA	resistance-nodulation-cell division (RND)	0.6306	1.0979	0.57	0.0071	unidentified *Sphingomonas*, unidentified *Massilia*, *Variovorax paradoxus*

Table S3. Potential risk chemicals in TSC and LSC. The potential risk chemicals are those that are classified under the hazard codes H300 to H336 according to the Globally Harmonized System (GHS) for indicating skin, eye, and airway irritation. The enriched risk chemicals in TSC and LSC were determined to be statistically significant ($p < 0.01$, FDR < 0.1, fold change > 2). Note that chemicals classified as drugs are excluded from this table.

group	Risk chemical category	Risk chemical	Molecular formula	GHS hazard statement	Symptom, disorder and disease	TSC/LSC fold changes	p-value
		Benfuracarb	$C_{20}H_{30}N_2O_5S$	H331: Toxic if inhaled	eye irritation	3.35	0.0004
		Carbofuran	$C_{12}H_{15}NO_3$	H330: Fatal if inhaled	abdominal pain; vomiting; skin irritation	>10	0.0002
		Famoxadone	$C_{22}H_{18}N_2O_4$		eye and skin irritant		
		Fenoxycarb	$C_{17}H_{19}NO_4$		headache, nausea, vomiting	>10	0.0006
TSC	pesticide/herbicide	Bromacil	$C_9H_{13}BrN_2O_2$	H315 (40.8%): Causes skin irritation<br>H319 (40.8%): Causes serious eye irritation<br>H335 (40.8%): May cause respiratory irritation	irritation eyes, skin, upper respiratory system; thyroid injury	2.74	0.0015

		Propazine	$C_9H_{16}ClN_5$		shortness of breath, muscle spasms, ataxia, and anorexia	3.16	0.0008
		Triethanolamine	$C_6H_{15}NO_3$	H315 (29.76%): Causes skin irritation<br>H318 (16.88%): Causes serious eye damage<br>H319 (82.38%): Causes serious eye irritation	atopic dermatitis	2.36	0.0004
		Diethanolamine	$C_4H_{11}NO_2$	H315: Causes skin irritation<br>H318: Causes serious eye damage	liver neoplasms;heart diseases;malformations of cortical development	2.81	0.0004
		m-Cresol	C_7H_8O	H311 (99.93%): Toxic in contact with skin<br>H314 (100%): Causes severe skin burns and eye damage	skin,eye irritation; vomiting	2.47	0.0004
		Diethyl phthalate	$C_{12}H_{14}O_4$		reproductive and developmental toxicity	3.84	0.0004
	detergents and cleaning products	o-Xylene	C_8H_{10}	H315 (100%): Causes skin irritation<br>H319 (29.81%): Causes serious eye irritation<br>H332 (100%): Harmful if inhaled<br>H335 (26.4%): May cause respiratory irritation	dermatitis; irritation; inflammation	3.55	0.0008
LSC	herbicide	Alachlor	$C_{14}H_{20}ClNO_2$	H317: May cause an allergic skin reaction	dermatitis; chronic inflammation	0.12	0.0004

Table S4. Potential protective metabolites in TSC and LSC. Flavonoids, isoflavonoids, indoles, and keto acids are characterized as protective metabolites for humans. The significantly enriched metabolites in these categories in TSC and LSC were determined to be statistically significant ($p < 0.01$, FDR < 0.1, fold change > 2). The molecular formula and features were obtained from PubChem. Metabolites annotated as synthetic drugs are excluded from this table.

Group	Protective metabolite category	Protective metabolite	Molecular formula	Feature	TSC/LSC fold changes	p-value
TSC	Indoles and derivatives	Indoleacetaldehyde	$C_{10}H_9NO$	intermediate metabolite in the metabolism of tryptophan	>2	0.0002
LSC	Indoles and derivatives	N-Acetylserotonin	$C_{12}H_{14}N_2O_2$	antioxidant	0.33	0.0004
	Flavonoids	Kaempferol	$C_{15}H_{10}O_6$	a plant metabolite, antioxidant	0.46	0.0022
		Genistein	$C_{15}H_{10}O_5$	a plant metabolite, antioxidant	0.25	0.0008
		6-Hydroxydaidzein	$C_{15}H_{10}O_5$	derived from daidzein, anti-inflammatory	0.29	0.0004
	Keto acids and derivatives	Oxoadipic acid	$C_6H_8O_5$	natural metabolic product found in bacteria and human	0.44	0.002
